# Thought on Food: A Systematic Review of Current Approaches and Challenges for Food Intake Detection

**DOI:** 10.3390/s22176443

**Published:** 2022-08-26

**Authors:** Paulo Alexandre Neves, João Simões, Ricardo Costa, Luís Pimenta, Norberto Jorge Gonçalves, Carlos Albuquerque, Carlos Cunha, Eftim Zdravevski, Petre Lameski, Nuno M. Garcia, Ivan Miguel Pires

**Affiliations:** 1 School of Technology, Polytechnic Institute of Castelo Branco, 6000-767 Castelo Branco, Portugal; 2 Escola de Ciências e Tecnologia, University of Trás-os-Montes e Alto Douro, Quinta de Prados, 5001-801 Vila Real, Portugal; 3 Health Sciences Research Unit: Nursing (UICISA: E), Nursing School of Coimbra (ESEnfC), 3046-851 Coimbra, Portugal; 4 Higher School of Health, Polytechnic Institute of Viseu, 3504-510 Viseu, Portugal; 5 Child Studies Research Center (CIEC), University of Minho, 4710-057 Braga, Portugal; 6 CISeD—Research Centre in Digital Services, Polytechnic Institute of Viseu, 3504-510 Viseu, Portugal; 7 Faculty of Computer Science and Engineering, University Ss Cyril and Methodius, 1000 Skopje, North Macedonia; 8 Instituto de Telecomunicações, Universidade da Beira Interior, 6200-001 Covilhã, Portugal

**Keywords:** food intake detection, biosensors, neural networks, image processing, nutrition

## Abstract

Nowadays, individuals have very stressful lifestyles, affecting their nutritional habits. In the early stages of life, teenagers begin to exhibit bad habits and inadequate nutrition. Likewise, other people with dementia, Alzheimer’s disease, or other conditions may not take food or medicine regularly. Therefore, the ability to monitor could be beneficial for them and for the doctors that can analyze the patterns of eating habits and their correlation with overall health. Many sensors help accurately detect food intake episodes, including electrogastrography, cameras, microphones, and inertial sensors. Accurate detection may provide better control to enable healthy nutrition habits. This paper presents a systematic review of the use of technology for food intake detection, focusing on the different sensors and methodologies used. The search was performed with a Natural Language Processing (NLP) framework that helps screen irrelevant studies while following the PRISMA methodology. It automatically searched and filtered the research studies in different databases, including PubMed, Springer, ACM, IEEE Xplore, MDPI, and Elsevier. Then, the manual analysis selected 30 papers based on the results of the framework for further analysis, which support the interest in using sensors for food intake detection and nutrition assessment. The mainly used sensors are cameras, inertial, and acoustic sensors that handle the recognition of food intake episodes with artificial intelligence techniques. This research identifies the most used sensors and data processing methodologies to detect food intake.

## 1. Introduction

The worldwide population has inadequate nutrition and physical activity habits, which are worst at a younger age [1,2]. It is causing different healthcare problems, including obesity, hypertension, and other diseases, mainly related to the digestive system [3,4]. These diseases are primarily associated with dietary problems that can be reduced with health literacy [5,6]. The nutritionists recommend a balanced diet to reduce different nutritional problems [7,8,9], which, with a busy lifestyle, can be challenging to obtain and difficult to monitor. It also helps to combat other problems in different countries related to malnutrition [10,11].

Mobile devices, such as smartphones and smartwatches, and other sensors, including electrogastrography, cameras, microphones, and inertial sensors, may help control nutrition habits with food intake detection and associated recommendations [12,13]. These sensors are commonly non-invasive, allowing their use in different environments and by different people [14,15] in their daily activities. The automatic detection and classification of eating habits can be also used to control the number of calories and the type of meals the individual has consumed, allowing the characterization of different habits and promoting the creation of a personalized system [16]. This subject is included in the developments related to Ambient Assisted Living and Enhanced Living Environments [17,18].

This paper consists of a systematic review of sensors and machine learning approaches for detecting food intake episodes. This research includes the use of different scientific databases regarding this subject. The various methods can provide different exciting results in the literature and pointers for further analysis.

The introductory section ends with this paragraph, and the remaining sections are organized as follows: Section 2 presents the methodology used for this systematic review, presenting the results in Section 3. After that, the results are discussed in Section 4, and the main conclusions are presented in Section 5.

## 2. Methodology

### 2.1. Food Intake Detection

Food intake is essential to survival, but more importantly, it is more an emotional act than one about survival. Eating is often related to emotions, and humans are reluctant to eat something that does not taste good to them [19]. As a result, the food industry has been trying to solve the equation of creating delicious food with the minimum cost possible. The answer was not the best one, with the advent of fast-food chains across the globe promising a meal that can be purchased, obtained, and consumed with very little time investment [20]. The busy lifestyle, with constant pressure to deliver more in less time, urges us to spend the shortest time possible on “non-productive” tasks.

To detect an excellent and healthy diet (or a bad one), one must identify the moment the person is eating. Only after detecting eating episodes can one face the challenge of detecting what food is consumed and taking measures to improve someone’s diet. This stage demands the use of appropriate sensors with advanced algorithms. As a result, the main goal of this research is to survey the current approaches for food intake detection, the first step towards a fully automated personalized diet experience.

### 2.2. Research Questions

The main questions of this systematic review were as follows: (RQ1) What sensors can be used to access food intake moments effectively? (RQ2) What can be done to integrate such sensors into daily lives seamlessly? (RQ3) What processing must be done to achieve good accuracy?

### 2.3. Inclusion Criteria

This paper studies different implementations of food intake detection using different sensors. The selection of different studies for this systematic review was performed with the following criteria: (1) research work that performs food intake detection; (2) research work that uses sensors to detect food with the help of sensors; (3) research work that presents some processing of food detection to propose diet; (4) research work that use wearable biosensors to detect food intake; (5) research work that use the methodology of deep learning, Support Vector Machines or Convolutional Neural Networks related to food intake; (6) research work that is not directly related to image processing techniques; (7) research work that is original; (8) papers published between 2010 and 2021; and (9) papers written in English.

### 2.4. Search Strategy

This research strategy follows a PRISMA (Preferred Items for Reporting Systematic Reviews and Meta-analyses) methodology [21] to identify and process the literature on food intake detection published between 2010 to 2021. Leveraging the NLP (Natural language Processing) toolkit, the following electronic databases were explored automatically for article selection: PubMed, Springer, ACM, IEEE Xplore, MDPI, and Elsevier.

The NLP framework input parameters use a collection of keywords to identify potentially relevant papers and a set of properties that should be satisfied by the identified papers. The following research keywords were used: “food intake detection” and “sensors” and “measurement”. Based on the DOI numbers, the program automatically eliminated all duplicates. Relevant papers identification is based on the initial keyword search and the inclusion criteria. The benefit of the framework is that many irrelevant articles can be quickly discarded by using robust searching methodologies such as stemming, fuzzy-matching, etc. As a result, the framework eliminates articles that are not original works (i.e., they are review articles, position papers, etc.) or are not relevant considering the research question. As a result, a significantly smaller subset of articles was obtained to only focus on selecting the articles to use in the qualitative synthesis. For more detailed information about the features of the NLP toolkit, more details are available in the study by Zdravevski et al. [22].

The authors independently evaluated every identified study, determining their suitability for inclusion in this paper. The studies were analyzed to identify the various methods for using sensors to detect food intake. The research was performed on 1 November 2021.

### 2.5. Extraction of Study Characteristics

Different data were extracted from the selected research papers. Table 1 presents other collected parameters in the following order: year of publication, the dataset used, purpose, sensors used, and study methodology. The source code and most datasets used in the analyzed papers are not publicly available.

## 3. Results

**Figure 1:**
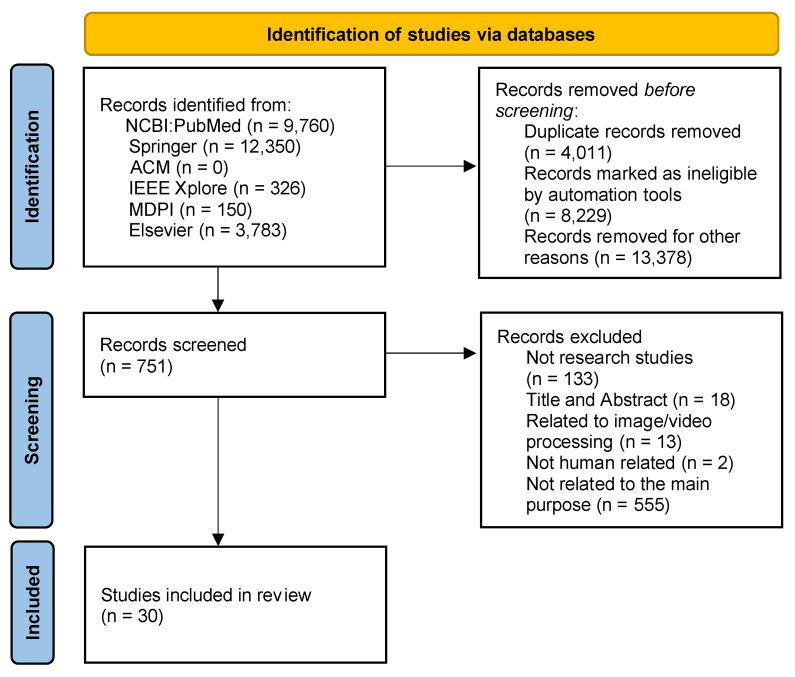
A flow diagram of the paper selection.

As presented in Figure 1, 26,369 papers were identified from the selected scientific databases. As this search used an NLP framework, 4011 papers were excluded as duplicated, 8229 were marked as ineligible, and 13,387 were removed by the automatic analysis of the metadata, resulting in 751 papers to be analyzed. After analyzing them by title and abstract, 133 papers were excluded by the study’s type and 18 by other keywords in the title and abstract. Next, the studies related to image/video processing were excluded, resulting in the exclusion of 13 papers. Following the remaining studies, 2 studies not associated with human analysis were also excluded, and 555 other studies were excluded after complete analysis because their purpose is not directly related to the main subject of this study. Finally, the remaining 30 research papers were synthesized and included in the qualitative and quantitative analysis.

### Presentation of the Selected Studies

Following the analysis of the 30 studies, the relevant data were extracted and presented in Table 1. The search performed for this systematic review consists of the finding of papers published between 2010, and 2021, where eight studies (27%) were published in 2021, six studies (20%) in 2020, seven studies (27%) in 2019, two studies (7%) in 2018, three studies (10%) in 2017, one study (3%) in 2016, one study (3%) in 2014, and two studies (7%) in 2016. Regarding the sensors used, seventeen studies (57%) used/acquired image/video data, eight studies (27%) used inertial sensors, eight studies (27%) used acoustic sensors, four studies (13%) used piezoelectric sensors, and other residual sensors were used, including electrocardiography sensors, electroglottography sensors, temperature, interbeat intervals, dermal activity, photoplethysmography, heart rate, and flex sensor.

Detecting chewing activities is challenging due to the daily movements of the head, mouth, and facial expressions. The studies analyzed in this paper differ in the sensors used, mainly falling into two categories: worn sensors and images captured of the room or the participant itself. In data processing, a pattern can be easily seen using neural networks in several forms, namely deep learning, for feature extraction. The dataset assumes particular importance in such scenarios, and some works provide too little information. This section presents an augmented approach to the information gathered.

Authors of [56] employ a wearable system based on a piezo-respiratory belt that converts changes in tension during breathing into a voltage signal. With this sensor, the authors present a structure to detect food and liquid intake through a person’s swallowing events. Dataset is unspecified primarily in this work, with authors stating that several signal segments were used from different human subjects on an SVM (Support Vector Machine) using a two-stage approach. In the first stage, the authors achieved a true positive rate higher than 82.9% and a false-positive lower than 1.9%. In the second stage, the accuracy ranged from 88% to 73.33%.

An SVM-based approach is also employed in [57], using a participant’s mobile device’s built-in camera to capture images fed to an SVM classifier using the RBF (Radial Basis Function) kernel. The participant uses the mobile device to photograph the plate before eating and at the end. The algorithm can extract features such as shape, color, size, and texture based on food image processing. The authors used a dataset of 200 images, half for training and half for testing, reaching an accuracy of around 92%. This accuracy is obtained using all image feature processing—color, texture, size, and shape. The authors provide detailed results for 30 food items using different features combined. The average using all features results in 92.21% accuracy.

A unique approach to food detection in terms of the sensor is presented in [55], using an electroglottograph (EGG) device, which detects the passage of food through electrical impedance variations on the larynx, helped by a PS3Eye camera to capture video of the participants. Another sensor was used in the form of a miniature throat microphone (MIC) placed over the laryngopharynx to capture swallow sounds. Data was collected through the participation of thirty individuals, with five left out. The experiment consisted of a 4-visit scenario involving the consumption of meals with self-selected content. Artificial Neural Networks were trained with subject-independent classifiers to identify periods of food intake from the wavelet features. The processes of training, validation, and testing were performed using the Neural network toolbox from Matlab R2011b. In terms of results for food intake recognition, leave-one-out cross-validation results showed average accuracies of 90.1% with a standard deviation of 8.5% for EGG and 83.1% with a standard deviation of 10.8% for the MIC model.

In what can be seen as the continuation of the previous work, in [54], a piezoelectric sensor system captures lower jaw motion and automatically measures chewing count and chewing rate. By placing a sensor under the participant’s ear, the vibration of the surface to which the sensor is attached creates strain within the piezo polymer material, generating a voltage. The dataset is very similar, with 30 participants with two different approaches in a total of 104 meals (16 were considered failed). Experiments were also captured on video with a Sony PS3Eye camera to validate time-synchronized sensor signals. Two approaches were used, a semi-automatic and a fully automatic method. In a semi-automatic process, histogram-based peak detection was used to count the number of chews in manually annotated chewing segments, resulting in a mean absolute error of 10.40% ± 7.03%. In the fully automatic approach, automatic food intake detection preceded the application of the chew counting algorithm. The sensor signal was divided into 5-s non-overlapping epochs. Chewing frequency was found to be in the range of 0.94 to 2 Hz, which with 5 s epochs can translate to multiple chewing and not chewing events inside a given epoch. Authors classify the epoch as chewing if at least half of the samples inside the epoch were considered as from chewing. However, this situation typically occurs only at the end of chewing sequences. Artificial neural network training was performed with a backpropagation algorithm on the three layers’ feed-forwarding architecture. The layers were defined as 38 neurons on the input layer for each feature, 5 neurons on the hidden (second) layer, and finally, the output layer with a single output neuron to indicate the predictor output class as chewing or not-chewing. Not chewing can be multiple events, such as the absence of chewing, rest, speech, and motion artifacts. Leave-one-out cross-validation was used to train an artificial neural network (ANN) to classify epochs as “food intake” or “no intake”, with an average F1-score of 91.09%. Chews were counted in epochs classified as food intake with a mean absolute error of 15.01% ± 11.06%.

A Recurrent Neural Network named SwallowNet is presented in [52]. Through a wearable necklace that comprises two piezoelectric sensors vertically positioned around the neck and an inertial motion unit with an accelerometer, gyroscope, and magnetometer, the authors calculate the number of swallows in food intake to detect the calorie intake of a person. Ten participants were used in this study that defined a recurrent neural network to see swallows in a continuous data stream after being trained purely from raw data using automated feature learning methods. An f-score of 76.07% versus 66.6% in the leave-one-out subject out cross-validation (LOSOCV) and a root mean square error (RMSE) of 3.34 in swallow count.

Authors of [51] present an approach using an acoustic doppler sonar for food intake monitoring, namely chewing and swallowing. The dataset comprised 10 participants with six different types of food, where the movement of the jaw and its vibration pattern differ depending on the type of food consumed. Using an artificial neural feature, extraction and classification are performed. The experimental results showed that the proposed method obtained maximum recognition rates of 91.4% and 78.4% for chewing and swallowing, respectively.

In [53] authors used an in-ear microphone to enable eating behavior monitoring. Using a 1-dimension convolutional neural network and 60 h of the semi-free-living dataset, the authors present results with only acoustic signal and fusion of acoustic and inertial sensors, leaving one subject out approach (fusion+ LOSO), thus enabling comparison. Results show that the presented approach with a 5-s input window achieves 0.89 precision and 0.92 recall, with 0.95 weighted accuracy, which proves to be better than fusion+.

To detect the ingestion sounds, namely swallowing and chewing, the authors of [49] used a throat microphone (iASUS NT3) using a Sony IC recorder at 44.1 KHz. The dataset comprised tracheal data recordings of 8 subjects (4 male and 4 female) between 22 and 29 years old. Authors used Convolutional Neural Networks to learn time-frequency features for food intake classification problems, define event detection systems and define spectrograms for food intake events. Experiments with a 2-fold cross-validation protocol achieved 0.792 precision, 0.752 recall, and 0.771 accuracy, which is higher than leave-one-subject-out.

By using a camera to identify the kinds and ingredients of food to determine whether a given diet is healthy, the authors of [50] present an approach based on a p-Faster R-CNN. They are using 300 types of Chinese food and 100 kinds of food in food-101 datasets, achieving an AP of over 0.7 in all considered food types. Authors compare results between faster R-CNN and p-Faster CNN in a tabular form, clearly proving the approach’s superiority in the specified scenarios.

The work presented in [42] aims the creation of an automatic ingestion monitor (AIM) using a neural network classifier. The AIM uses a hand gesture sensor on the dominant hand, a piezoelectric strain sensor, and a data collection module. The system captured data from 40 participants using a neural network classifier. Results presented in the paper state that, for activity annotation, the raters achieved an average *kappa* value of 0.74 with a standard deviation of 0.02 and for food intake annotation average *kappa* was 0.82 with a standard deviation of 0.04. *Kappa* was defined as Cohen’s *kappa*-based inter-rater reliability testing as presented in Equation (1).

(1) $$kappa=\frac{Prob\left(a\right)-Prob\left(e\right)}{1-Prob\left(e\right)}$$

The *Prob(a)* and *Prob(e)* values represent the probability of observed and expected agreement, respectively. *Kappa* results in a value between −1 and 1, where zero or negative value denotes no agreement, a value between 0.6 and 0.8 satisfactory agreement, and over 0.8 indicate perfect agreement.

In [43], the authors present a prototype wearable food intake monitoring system consisting of a wrist band and an upper arm band, with 9-axis inertial motion sensors through an accelerometer, magnetometer, and gyroscope. The dataset comprised 25 min of data divided into 30 s segments while eating, shaving, and brushing teeth. The authors used machine learning to reduce false-positive eating detection after using a Kalman filter to detect the position of the hand relative to the mouth.

An approach based on video capture is presented in [44], where 85 videos with people eating from a side-view perspective were used. Authors extract human motion features from the recorded video sequences through a deep network. A two-stream deep network is proposed to process body and face motion, together with the first deep network to take advantage of both features. Experimental results show an f-score of 0.9173, with a precision of 0.9175 and a recall of 0.9171.

An approach for glycemic index regulation through the calculation of food size, swallowing style, and consumption time can be found in [45]. The authors used a dataset from 30 diabetic persons to confirm glucose levels with a glucometer. Using MEMS technology, an acoustic sensor is placed on the trachea, and data is fed to a deep belief network with Belief Net and Restricted Boltzmann Machine combined. The authors searched for mastication level analysis, detecting chewing and swallowing, without chewing and drinking, and finally only saliva swallow, presenting signal graphs of each occurrence type. The authors presented various analyses of different signals collected while the subject is chewing different kinds of food. Among other things, they provide the signal waveform of the acoustic signal produced while eating 10 g of solid food for 10 s. The authors proved that chewing and swallowing styles can affect glycemic index in participants with more than four years of the diabetic condition.

In [46], an image-based approach for food image detection and recognition for Korean food is presented. The dataset comprised 4000 images obtained from restaurants and internet searches. The authors used a digital camera to capture images fed into a deep learning convolutional neural network—K-foodNet. The training process used TensorFlow with a batch size of 64. Results of K-foodNet point to a 91.3% accuracy and a prediction time of 0.42 ms, which, compared to other approaches, fares very favorably. The authors also present results for AlexaNet, GoogleNet, VGG-19, and ResNet-18 in a table.

The authors of [47] presented a dietary intake on shared food scenarios, though detection of the subject’s face, hands, and food based on images. A dataset of 360 videos and a COCO dataset to train a mask R-CNN. R-CNN detects food class, the bounding box indicating each food item’s location, and the segmentation mask. The authors argued that it can be possible to calculate food volume based on food masks. Results are presented for two scenarios—2 participants sharing a pizza and 3 participants sharing multiple food items. A table is presented with results, with the authors considering them satisfactory.

The paper [48] presents an approach based on a camera mounted on a glasses frame named Automatic Ingestion Monitor 2.0. Two datasets of 1600 images each were used, one with the presence of food and another without any food. The system aims to minimize the number of analyzed images needed to be processed either by a human operator or a computer vision algorithm for food image analysis. Several image processing techniques were used: lens barrel distortion, image sharpness analysis, and face detection blurring.

A mixed reality headset with cameras is used to detect diet-related activities and support healthy choices proposed in [34]. Using automatic vending machines, the authors used 10,035 labeled product image instances of their creation in a real-world environment. A comparison of several approaches based on neural networks is presented with the associated results. The authors concluded that MR headsets can be effectively used in an Internet-of-People scenario that helps the user make healthier food choices more effectively than a smartphone-based approach.

In [35], the authors presented a different approach in terms of sensors to capture data by employing an Electrocardiogram (ECG) device to register heart rate variability. Two datasets were used, the first with 16 participants to train the artificial neural network with the leave-one-subject-out method, and the second with ECG recordings from 37 healthy control participants and 73 patients with functional dyspepsia. The authors experimented with two major cross-validation approaches—leave-one-subject-out and leave-one-subject-out leave-one-out (LOSO-LOO). For LOSO, the mean accuracy was 0.83, the mean sensitivity was 0.51, and the mean specificity was 0.89. With LOSO-LOO, the ANN reached maximal accuracy with 2-min epochs at 0.93 and 0.79 and a mean sensitivity of 0.79. Also, the mean specificity increased to 0.97.

Another image-based approach for food detection can be found in [36], using the AIM 2.0 monitor with the CNN-based image classifier implemented on the CortexM7 controller instead of a computer. The dataset comprised 15,343 images (2127 food and 12,216 not food). A detailed description of the implementation of the CNN-based image classifier in the CortexM7 is given in the paper. The proposed model achieves an accuracy of 75% and an F-score of 74% in testing, demonstrating great promise in real-time image classification.

The main objective of [38] is to design and develop a GUI-based interactive tool capable of identifying the type of food with good efficiency. The authors achieved 96.81% accuracy using a CNN for image classification and detection. The dataset Food101 is used, with 101,000 images of 101 food categories. Images are captured by the user using a mobile phone camera and fed to the system for classification.

In another approach of a glasses-mounted camera, the authors of [37] present a system that can not only monitor but also create user awareness about how much food is too much. The iLog system provides information on a person’s emotional state and the classification of eating behaviors from everyday eating to stress-eating. The model was trained with 800 images and tested with 200. The iLog model has produced an overall accuracy of 98% with an average precision of 85.8%. The quantified foods are then compared to the stored database of the user nutrition in Firebase, providing feedback using a mobile application interface.

A mobile device camera is also used in [40], with a mask R-CNN for smart food recognition and automatic dietary assessment. The authors used the COCO2017 dataset with several food items. Results include classification error rates for ten types of food. Error rates range from 0.23 for a sandwich to 9.86 for broccoli. The authors also presented classification accuracy based on the plate contents—fast food, fruit, salad, and dessert, with an average value of 0.875.

The work of [23] presents a multiple sensor array system with temperature, interbeat intervals, dermal activity, photoplethysmography, heart rate (on the first dataset), and a wristband 9-axis inertial motion measurement units (using the second dataset). The focus is on data fusion using a deep residual network to gain a more comprehensive insight into human activity dynamics. The authors considered the statistical dependency of multisensory data and explored intramodality correlation patterns for different activities. In terms of the dataset, two scenarios were considered. The first shows data from three days of wristband device use by a single person. The second is an open dataset of 10 individuals performing 186 activities (mobility, eating, personal hygiene, and housework). A comprehensive table is presented with results of the deep learning classifier performance with an F1-score of 0.80335, an accuracy of 0.95083, and a precision of 0.80355.

In [24], an array of sensors is mounted on a glasses frame—a camera, accelerometer, and flex sensor in contact with the temporalis muscle. The dataset comprises 30 volunteers using the system for 24 h in pseudo-free living and 24 h in a free-living environment. The authors used an SVM model to detect food intake through data fusion between the accelerometer and flex sensor. The camera was used to capture data every 15 s and validate sensor fusion decisions, thus was not used on the SVM model. The AIM-2 detected food intake over 10-s epochs with a (mean and standard deviation) F1-score of 81.8 ± 10.1%. The accuracy of eating episode detection was 82.7%.

In [25], the authors used data fusion for automatic food intake gesture detection, but without any sensors. The paper focuses on processing video and inertial data using deep learning, with a dataset of 100 participants consuming food in discrete portions (OREBA-DIS) and 102 participants while consuming a communal dish (OREBA-SHA). The authors employed a fusion of inertial and video data with several deep learning techniques. On the OREBA-DIS dataset, the max score fusion approach obtained an F1 of 0.871, while individual video obtained an F1-Score of 0.855 and inertial even lower with an F1-Score of 0.806. However, with the OREBA-SHA dataset, the max score fusion approach only obtained an F1-Score of 0.873, while the individual inertial model obtained 0.895. Pairwise comparisons using bootstrapped samples confirm the statistical significance of these differences in model performance were conducted with pairwise comparisons using bootstrapped samples, resulting in *p* < 0.001.

An approach based on inertial sensors present on some smartwatches—accelerometer and gyroscope can be found in [27]. The authors employed CNN for feature extraction and an LSTM network to model temporal evolution. Both parts are jointly trained by minimizing a single loss function. The FIC, FreeFIC, and FreeFIC held-out datasets contain data related to triaxial acceleration and orientation velocity signals. The authors presented a complete framework for automated modeling of in-meal eating behavior and temporal localization of meals. Results are presented for both datasets, FIC and FreeFIC, in a tabular form with time in seconds for mean, standard deviation, median, total, and the total number of hours in terms of meal sessions and food intake cycles.

Lee et al. [30] presented an approach based on ultrasonic doppler shifts to detect chewing events and a camera placed on the user’s neck to capture images. Eight participants were involved in noise environments, and a Markov hidden model recognizer was used to maximize swallow detection accuracy. A linear regression model was also used to find a relation between chewing counts and food intake. CNN was used for feature extraction to recognize food items based on the camera images. Results are presented based on the mean absolute percentage error metric, with signal-to-noise ratio information on several scenarios in tabular form.

A body area network of sensors is presented in [31], encompassing a camera on the user’s chest with a system hub, phones with an added microphone and dedicated hardware to capture chewing and swallowing sounds, and a wrist-worn band with an accelerometer and gyroscope. Emphasis is given to the system hardware and data acquisition, not data processing. The system was provided to some students, and some pictures of them using the prototype are on the paper.

In [32], the authors present a custom earbud with two microphones—one in-ear and the other external to gain insight on dietary activity, namely chews per minute and causes for food choices. A total of 6 participants used the system for 6 h in 59 h of collected data. The processing uses a deep neural network and multiple Gaussian processes per feature to perform multiclass classification. Regarding results, on laboratory data with ground truth, chewing detection recall was 84%, intake 78%, and drinking 88%.

Finally, in [33], the authors used video and inertial sensor data with two datasets of annotated intake gestures—OREBA and Clemson. The work aims to present a single-stage approach that directly decodes the probabilities learned from sensor data into sparse intake detection for eating and drinking. A deep neural network with weakly supervised training using Connectionist Temporal Classification loss and decoding using an extended prefix beam search decoding algorithm. The single-stage models present improvements of 3.3% and 17.7% over the author’s implementations of SOTA for inertial and video modalities, respectively.

## 4. Discussion

### 4.1. Interpretation of the Results

**Figure 2:**
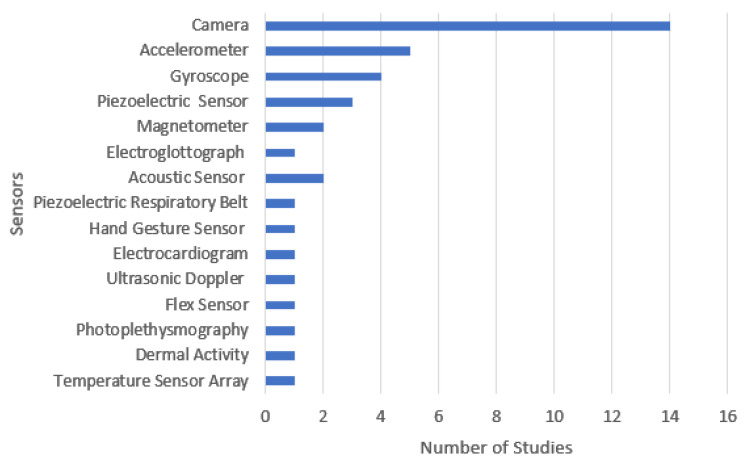
The relation between sensors and the number of studies.

As seen from the literature analysis presented in Section 3, most studies use a two-layer approach—a sensor array to capture data and a neural network-based processing scheme. Three major categories were identified in terms of sensors (Figure 2 presents the sensors used in the literature surveyed)—cameras, acoustic and inertial. Cameras detect food contents and food intake, acoustic sensors capture chewing sounds, and inertial sensors capture positional data. Cameras are studied in two scenarios: user-worn and room surveillance. The latter can prove to be very intimidating and costly since it should be placed in every room where the user uses to eat. However, it can monitor several people’s eating without needing individual sensors, such as for several family members in a shared food scenario. Regarding placement, acoustic and inertial sensors are typically body-worn devices, whereas some workers used microphones on the participants’ necks to detect chewing sounds. Inertial sensors, such as those in a smartwatch, can provide meaningful information about the participant’s hand position and gesture recognition. Finally, some research works go even as far as fusing data from multiple categories.

Focusing on the selected studies, Figure 2 presents the number of studies using different sensors. One can see the dominance of cameras, mainly because convolutional neural networks work very well with feature extraction over images, and cameras are easy to integrate and obtain, namely using a smartphone and asking the user to take a picture of the plate. However, in such a scenario, the system is still dependent on user input, which may lead to the user forgetting to take pictures of everything he eats.

**Figure 3:**
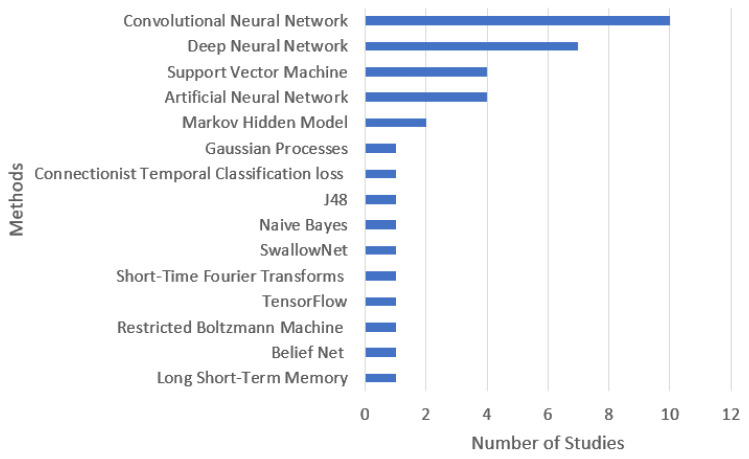
The relation between methodology and the number of studies.

Regarding the methods explored (Figure 3), convolutional neural networks are the primary and most implemented method (ten studies). The second most used method is deep neural networks (seven studies), followed by support vector machines and artificial neural networks (four studies) and Markov hidden models (2 studies).

### 4.2. Comparison of the Different Studies Analyzed

Table 2 shows the relationship between the sensors employed and the methodology used for the studies. This table was created by analyzing each article and identifying which sensors were explicitly mentioned in the study and which methods were applied. The heterogeneity of the approaches clearly states that this is still a research topic for which standard approaches do not exist. The coexistence of multiple sensor data streams can be beneficial by using data fusion, but only if the user is present with a seamless solution. As stated previously, in terms of sensors placed on the participant, comfort (and convenience) must prioritize to motivate its use.

If there seems to be discord on sensors, in terms of methodologies, the span is not so great. Neural network-based approaches dominate, which is the current trend in many research areas. Deep learning, convolutional neural networks, and support vector machines are the most used methods.

### 4.3. Answers to Identified Research Questions

There are multiple advantages to using sensors for food intake detection. These days, people have increased their attention regarding nutritious and healthy living due to the recent pandemic crisis that forced increased time spent at home. Calories and other nutrients can be tracked by logging the type and weight of food intake and further processing these data. However, sensors are essential in overcoming the error and intrusiveness of a food logging system in the user routine.

The main findings from the 34 studies identified by this review are as follows. Concerning RQ1, “What sensors can be used to access food intake moments effectively?” sensors explored in the studies analyzed fall into the three categories: camera, inertial and acoustic type. Integrating data acquired from several sensors can improve the accuracy of individual sensors [23,25]. One can also equate using fixed cameras on a stationary scenario in a room. However, the authors of this study believe that cameras are more useful for nutrient and meal size detection than food intake detection, which is a topic out of the scope of this article. A plate picture does not imply that the participant will ingest all that food and thus incur imprecisions. Inertial sensors can effectively detect if a person is eating by analyzing data from a wristband. Acoustic sensors present a challenge in placement, which may be critical for performance, with works focusing on neck positioning to increase sensibility.

Regarding RQ2, “What can be done in terms of seamless integration of such sensors in daily lives?”, a system must be devised that integrates such sensors in a manner that the user feels at ease with. Substituting a watch for a smartwatch can be an easy adaptation but placing sensors in the neck is not so much. Certain works further try adapting glasses frames to include sensors [24,37,48]. The authors of this study consider that comfort must be the main design criteria for the sensor array. A user may easily consider using a wristband or a smartwatch to an entire sensor array with a central unit, camera, wristband and acoustic sensor tied to a necklace.

Finally, related to RQ3, “What processing must be done to achieve a good accuracy?”, the data gathering process must be easy to reproduce. Regarding the smartwatch example and heart rate sensor, manufacturers present an ideal position of the watch to increase sensor effectiveness. Such a scenario can also be present in these works, namely when considering taking pictures and capturing sounds. Calibration can also be needed (factory or user-initiated). As a result, data capture itself presents a challenge. Considering the data is good enough, processing points to neural networks-based approaches, as shown previously. Most works rely on a personal computer or cloud-based computing for processing, but at least one is running the processing on a mobile phone [57]. Strangely enough, it is also one of the oldest works analyzed in this study, so a possibility for further processing, namely using TensorFlow Lite by Google for mobile and edge devices.

### 4.4. Research Opportunities

The works identified to focus on sensors and data processing but provide no insights into participant emotions. Many studies are done in a controlled environment in a lab or a room, where participants are invited to consume one or more meals while wearing devices. One of the significant research opportunities can be the perceived acceptance of the participants in wearing such systems daily. Comfort must be the primary design criteria for these sensor systems, or they will fail.

In terms of using cameras, how does the participant feel about being surveyed regarding their daily activities? Privacy is more of a concern in the digital world, so that studies could be done on user perception on these sensitive matters.

Many works employ machine learning, deep learning, and neural networks. Although the results are auspicious, no approach reaches 100% accuracy. What can be added to the current strategies to increase accuracy or automatically detect false positives and negatives? Sensor fusion has already been explored, but even so, the 100% mark is unachievable.

## 5. Conclusions

This article has systematically reviewed sensors and automated approaches for detecting food intake episodes. A total of 30 papers matched the inclusion criteria and were included in the quantitative and qualitative analysis. The existence of these exciting papers shows that the use of sensors for the detection of food intake episodes is an exciting field. However, this research is currently open, and new opportunities can be developed around it to help people to have good nutrition and physical activity habits.

This review highlights the most used sensors and detection methodologies, including artificial intelligence techniques based on previous developments. In future work, the use of the sensors must be explored, and a mobile system for detecting food episodes may help individuals have rules to maintain a healthy nutrition lifestyle.

Is the food detection topic closed? As the results show, the answer is complex, it is still a very ongoing subject, and no work attained 100% accuracy. However, the authors are going further to achieve a personalized nutrition experience. More than detecting food intake, they aim to detect what is effectively ingested and its influence on health. Many challenges arise from this primary goal, from data gathering to processing, storage and retrieval, nutritional model, and motivation.

## Figures and Tables

**Table 1:** The study analysis.

Paper	Year of Publication	Population/Dataset	Purpose of Study	Sensors Used	Methodology
Bahador et al. [23]	2021	Two scenarios: 1. data from three days of wristband device use form a single person, and 2. Open data set of 10 individuals performing 186 activities (mobility, eating, personal hygiene, and housework)	Develop a data fusion technique to achieve a more comprehensive insight of human activity dynamics. Authors considered statistical dependency of multisensory data and exploring intramodality correlation patters for different activities.	Sensor array with temperature, interbeat intervals, dermal activity, photoplethysmography, heart rate (1st dataset). Wristband 9 axis inertial measurement units (2nd dataset)	Deep residual network.
Doulah et al. [24]	2021	30 volunteers using the system for 24 h in pseudo-free-living and 24 in a free-living environment	Food intake detection, sensor fusion classifier (accelerometer and flex sensor). Image sensor was used to capture data every 15 s and validate sensor fusion decision.	5 mp camera glasses add-on, accelerometer and flex sensor in contact with temporalis muscle	SVM model.
Heydarian et al. [25]	2021	OREBA dataset [26], composed by OREBA-DIS with 100 participants consuming food in discrete portions and OREBA-SHA with 102 participants while consuming a communal dish	Data fusion for automatic food intake gesture detection	Although no sensors were used, dataset was obtained through video and inertial sensors data	Fusion of inertial and video data with several methods that use deep learning.
Kyritsis et al. [27]	2021	FIC [28], FreeFIC [29], and FreeFIC held-out datasets containing triaxial acceleration and orientation velocity signals	A complete Framework towards automated modeling of in-meal eating behavior and temporal localization of meals	Data from smartwatch either worn on right or left wrist—accelerometer and gyroscope	CNN for feature extraction and LSTM network to model temporal evolution. Both parts are jointly trained by minimizing a single loss function.
Lee [30]	2021	8 participants in noisy environments	Detect eating events and calculate calorie intake	Ultrasonic doppler shifts to detect chewing events and a camera placed on user’s neck	Markov hidden model recognizer to maximize swallow detection accuracy. Relation between chewing counts and amount of food through a linear regression model. CNN to recognize food items.
Mamud et al. [31]	2021	Not specified, students were used with emphasis on acoustic signal	Develop a Body Area Network for automatic dietary monitoring system to detect food type and volume, nutritional benefit and eating behavior	Camera on chest with system hub, phones with added microphone and dedicated hardware to capture chewing and swallowing sounds, wrist-worn band with accelerometer and gyroscope	Emphasis was given to the hardware system and the captured signals, but not on signal processing itself.
Mirtchouk and Kleinberg [32]	2021	6 subjects for 6 h in a total of 59 h of data	Gain insight on dietary activity, namely chews per minute and causes for food choices	Custom earbud with 2 microphones—one in-ear and one external	SVDKL uses a deep neural network and multiple Gaussian Processes, one per feature, to do multiclass classification.
Rouast and Adam [33]	2021	Two datasets of annotated intake gestures—OREBA [26] and Clemson University	A single stage approach which directly decodes the probabilities learned from sensor data into sparse intake detection—eating and drinking	Video and inertial data	Deep neural network with weakly supervised training using Connectionist Temporal Classification loss and decoding using an extended prefix beam search decoding algorithm.
Fuchs et al. [34]	2020	10,035 labeled product image instances created by the authors	Detection of diet related activities to support health food choices	Mixed reality headset-mounted cameras	Comparison of several neural networks were performed based on object detection and classification accuracy.
Heremans et al. [35]	2020	16 subjects for training, and 37 healthy control subjects and 73 patients with functional dyspepsia for testing	Automatic food intake detection through dynamic analysis of heart rate variability	Electrocardiogram	ANN with leave-one-out.
Hossain et al. [36]	2020	15,343 images (2127 food images and 13,216 not food images)	Target and classify images as food/not food	Wearable egocentric camera	CNN based image classifier in a Cortex M7 microcontroller.
Rachakonda et al. [37]	2020	1000 images obtained from copyright-free sources—800 used for training and 200 for testing	Focus on eating behavior of users, detect normal eating and stress eating, create awareness about its food intake behaviors	Camera mounted on glasses	Machine learning models to automatically classify the food from the plate, automatic object detection from plate, and automatic calorie quantification.
Sundarramurthi et al. [38]	2020	Food101 dataset [39] (101,000 images with 101 food categories)	Develop a GUI-based interactive tool	Mobile device camera	Convolutional Neural Network for food image classification and detection.
Ye et al. [40]	2020	COCO2017 dataset [41]	A method for food smart recognition and automatic dietary assessment on a mobile device	Mobile device camera	Mask R-CNN.
Farooq et al. [42]	2019	40 participants	Create an automatic ingestion monitor	Automatic ingestion monitor—hand gesture sensor used on the dominant hand, piezoelectric strain sensor, and a data collection module	Neural network classifier.
Johnson et al. [43]	2019	25 min of data divided into 30 s segments, while eating, shaving, and brushing teeth	Development of a wearable sensor system for detection of food consumption	Two wireless battery-powered sensor assemblies, each with sensors on the wrist and upper arm. Each unit has 9-axis inertial measurement units with accelerometer, magnetometer, and gyroscope	Machine learning to reduce false positive eating detection after the use of a Kalman filter to detect position of hand relative to the mouth.
Konstantinidis et al. [44]	2019	85 videos with people eating from a side view	Detect food bite instances accurately, robustly, and automatically	Cameras to capture body and face motion videos	Deep network to extract human motion features from video sequences. A two-steam deep network is proposed to process body and face motion, together with the data form the first deep network to take advantage of both types of features simultaneously.
Kumari et al. [45]	2019	30 diabetic persons to confirm glucose levels with a glucometer	Regulate glycemic index through calculation of food size, chewing style and swallow time	Acoustic sensor in trachea using MEMS technology	Deep belief network with Belief Net and Restricted Boltzmann Machine combined.
Park et al. [46]	2019	4000 food images by taking pictures of dishes in restaurants and Internet search	Develop Korean food image detection and recognition model for use in mobile devices for accurate estimation of dietary intake	Camera	Training with TensorFlow machine learning framework with a batch size of 64. Authors present a deep convolutional neural network—K-foodNet.
Qiu et al. [47]	2019	360 videos and COCO dataset to train mask R-CNN	Dietary intake on shared food scenarios—detection of subject’s face, hands and food	Video camera (Samsung gear 360)	Mask R-CNN to detect food class, bounding box indicating the location and segmentation mask of each food item. Predicted food masks could presumably be used to calculate food volume.
Raju et al. [48]	2019	Two datasets (food and no food) with 1600 images each	Minimization of number of images needed to be processed either by human or computer vision algorithm for food image analysis	Automatic Ingestion Monitor 2.0 with camera mounted on glasses frame	Image processing techniques—lens barrel distortion, image sharpness analysis, and face detection and blurring.
Turan et al. [49]	2018	O participants, 4 male and 4 female, 22–29 years old	Detection of ingestion sounds, namely swallowing and chewing	Throat microphone with IC recorder	Captured sounds are transformed into spectrograms using short-time Fourier transforms and use Convolutional Neural network for food intake classification problem.
Wan et al. [50]	2018	300 types of Chinese food and 101 kinds of western food from food-101	Identify the ingredients of the food to determine if diet is healthy	Digital camera	p-faster R-CNN based on Faster-CNN with Zeiler and Fergus model and Caffe network.
Lee [51]	2017	10 participants with 6 types of food	Food intake monitoring, estimating the processes of chewing and swallowing	Acoustic Doppler sonar	Analysis of the jaw and its vibration pattern depending on type of food, feature extraction and classification with an Artificial Neural Network.
Nguyen et al. [52]	2017	10 participants in a lab environment	Calculate the number of swallows in food intake to calculate caloric values	Wearable necklace with piezoelectric sensors, accelerometer, gyroscope and magnetometer	A recurrent neural network framework, named SwallowNet, detects swallows on continuous data steam after being trained with raw data using automated feature learning methods.
Papapanagiotou et al. [53]	2017	60 h semi-free living dataset	Design a convolutional neural network for chewing detection	In-ear microphone	1-dimensional convolutional neural network. Authors also present results from leave-one-subject-out with fusion+ (acoustic and inertial sensors)
Farooq et al. [54]	2016	120 meals, 4 visits of 30 participants, from which 104 meals were analyzed	Automatic measurement of chewing count and chewing rate	Piezoelectric sensor to capture lower jaw motion	ANN machine learning to classify epochs as chewing or not chewing. Epochs were derived from sensor data processing.
Farooq et al. [55]	2014	30 subjects (5 were left out) in a 4-visit experiment	Automatic detection of food intake	Electroglottograph, PS3Eye camera and miniature throat microphone	Three-layer feed-forward neural network trained by the back propagation algorithm, neural network toolbox of Matlab.
Dong et al. [56]	2013	3 subjects, one female and two males	Development of a system for wireless and wearable diet monitoring system to detect solid and liquid swallow events based on breathing cycles	Piezoelectric respiratory belt	Machine learning for feature extraction and selection.
Pouladzadeh et al. [57]	2013	Over 200 images of food, 100 for training set and another 100 for testing set	Measurement and record of food calorie intake	Built-in camera of mobile device	Image processing using color segmentation, k-means clustering and texture segmentation to separate food items. Food portion identification through SVM and calorific value of food using nutritional table.

**Table 2:** The relation between methodologies and the sensors used.

	Sensors															Methodology														
Studies	Accelerometer	Gyroscope	Piezoelectric Strain Sensor	Magnetometer	Electroglottograph	Camera	Acoustic Sensor	Piezoelectric Respiratory Belt	Hand Gesture Sensor	Electrocardiogram	Ultrasonic Doppler	Flex Sensor	Photoplethysmography	Dermal Activity	Temperature Sensor Array	Convolutional Neural Network	Deep Neural Network	Support Vector Machine	Artificial Neural Network	J48	Naive Bayes	Swallow Net	Short-Time Fourier Transforms	TensorFlow	Restricted Boltzmann Machine	Belief Net	Markov Hidden Model	Long Short-Term Memory	Gaussian Processes	Connectionist Temporal Classification Loss
Bahador et al. [23]													X	X	X		X													
Doulah et al. [24]	X					X						X						X												
Heydarian et al. [25]						X											X													
Kyritsis et al. [27]	X	X														X												X		X
Lee [30]											X					X											X			
Mamud et al. [31]	X	X					X																							
Mirtchouk and Kleinberg [32]							X										X												X	
Rouast and Adam [33]						X											X													
Fuchs et al. [34]						X	X																							
Heremans et al. [35]										X									X											
Hossain et al. [36]						X										X														
Rachakonda et al. [37]						X																								
Sundarramurthi et al. [38]						X										X														
Ye et al. [40]						X										X														
Farooq et al. [42]			X						X								X													
Johnson et al. [43]	X	X		X														X												
Konstantinidis et al. [44]						X											X													
Kumari et al. [45]							X																		X	X				
Park et al. [46]																X								X						
Qiu et al. [47]						X										X														
Raju et al. [48]						X																								
Turan et al. [49]							X									X							X							
Wan et al. [50]						X										X														
Lee [51]							X																							
Nguyen et al. [52]	X	X	X	X																		X								
Papapanagiotou et al. [53]						X	X									X														
Farooq et al. [54]			X																X											
Farooq et al. [55]					X	X											X		X								X			
Dong et al. [56]								X										X		X	X									
Pouladzadeh et al. [57]						X												X												

## Data Availability

Not applicable.
